# Racially and Ethnically Diverse Sexual and Gender Minority Dementia Caregivers: Findings from the Qcare Study

**DOI:** 10.1002/alz70858_104385

**Published:** 2025-12-26

**Authors:** Krystal R. Kittle, Jason D. Flatt, Joel G Anderson, Mariana Pinto Alvarez

**Affiliations:** ^1^ University of Massachusetts‐Amherst, Amherst, MA, USA; ^2^ University of Nevada Las Vegas School of Public Health, Las Vegas, NV, USA; ^3^ University of Tennessee, Knoxville, TN, USA; ^4^ College of Nursing, University of Tennessee, Knoxville, TN, USA; ^5^ University of Massachusetts Amherst, Amherst, MA, USA

## Abstract

**Background:**

Caregivers of people with dementia endure unique emotional, physical, social, and financial challenges related to the care they provide and are often regarded as ‘invisible second patients.’ Akin to the invisible dementia caregiver, sexual and gender minority (SGM) dementia caregivers, or those who identify as lesbian, gay, bisexual, transgender, queer, nonbinary, intersex, or who express identities, behaviors and/or attractions that do not align with heterosexual or conventional gender norms, are underrepresented in dementia caregiving research, policy, and practice, deeming them especially invisible.

**Method:**

We conducted in‐depth interviews (*n* = 15) to explore the lived experiences of racially and ethnically diverse SGM dementia caregivers. Using content analysis, we identified overarching themes that represented socioenvironmental, risk and protective factors thought to influence the health and well‐being of racially and ethnically diverse SGM dementia caregivers.

**Result:**

Most participants identified as Black/African American (*n* = 8). Additional identities included Chinese, Native Hawaiian, Hispanic, Native American, or mixed race. In terms of gender identity, 9 identified as women, 3 men, 1 non‐binary, 1 transgender man, and 1 as two‐spirit. We identified four initial themes: (1) Socioenvironmental factors (Caregiver status, SGM identity, Racial/ethnic identity); (2) Risk factors (Gender identity/sexual orientation‐based discrimination, Lack of external support, SGM spaces, Racial discrimination); (3) Protective factors (Education, Self‐protective behaviors, Social support); and (4) Health and wellbeing (Dementia symptoms, Physical health, Mental health). Preliminary findings of note come from underrepresented caregivers (bisexual, pansexual, queer, transgender and non‐binary caregivers), who described the added stress (in addition to caregiving) caused by their care recipient's refusal to accept their sexual and/or gender identities, discomfort in discussing their caregiving experiences with their peers in the SGM community, and discriminatory behaviors from health care providers directed at them and their care recipients.

**Conclusion:**

While this research is ongoing, preliminary findings highlight factors worth considering when establishing targeted, effective interventions to improve the health and well‐being of racially and ethnically diverse SGM dementia caregivers. Moreover, these findings elucidate new areas that should be considered in future research related to the caregiving experiences and health of diverse SGM dementia caregivers.

